# Open Glenoid Augmentation With Scapular Spine Autograft for Recurrent Shoulder Instability: A Report of Two Cases

**DOI:** 10.7759/cureus.33654

**Published:** 2023-01-11

**Authors:** Rafat H Solaiman, Trenton Cooper, Bradley Nelson, Marc A Tompkins

**Affiliations:** 1 Department of Orthopedic Surgery, University of Minnesota Medical School, Minneapolis, USA; 2 Department of Orthopedic Surgery, Gillette Children's Specialty Healthcare, Saint Paul, USA

**Keywords:** arthroscopy, scapular spine autograft, open glenoid augmentation, glenoid bone defect, shoulder instability, shoulder dislocation

## Abstract

Optimal treatment for patients with significant glenoid bone loss after severe shoulder dislocation remains a topic of discussion, as there are many autograft and allograft techniques for glenoid augmentation. Several studies have identified scapular spine autograft to be a potential option for restoring glenohumeral stability, however, there is limited clinical data for this procedure. We present two cases in which patients suffered from anterior glenoid bone loss and recurrent shoulder instability who underwent open glenoid augmentation with scapular spine autograft. Both patients report a full return to activity with no functional limitations. Open glenoid augmentation with a scapular spine autograft is a viable option for patients with anterior glenoid bone loss and recurrent shoulder instability.

## Introduction

Anterior shoulder dislocation is a common shoulder injury and can progress to recurrent shoulder instability. Severe or recurrent dislocations can cause osseous defects of the glenohumeral joint which complicates stabilization procedures [1]. Arthroscopic Bankart repair is a common procedure for treating anterior shoulder instability; however, studies have indicated failure as high as two-thirds of the time in patients with significant humeral or glenoid bone loss [2]. Optimal treatment for these patients with significant bone loss remains a topic of discussion with a wide array of autograft and allograft techniques utilized for glenoid augmentation. The Latarjet procedure, using coracoid autografts, is perhaps most commonly used to restore glenoid bone loss; however, high complication rates of coracoid grafts have urged the exploration of other graft options [3]. Several studies have identified scapular spine autograft to be suitable for restoring glenohumeral stability from bone quality and biomechanical standpoints; however, there are few reports on the indications and details of this procedure [4-6].

We present two patients with anterior glenoid bone loss who successfully underwent open glenoid augmentation with scapular spine autograft for recurrent shoulder instability. Both patients were asked for permission to submit data concerning their cases for publication, and they provided consent.

## Case presentation

Case 1

**Figure 1:**
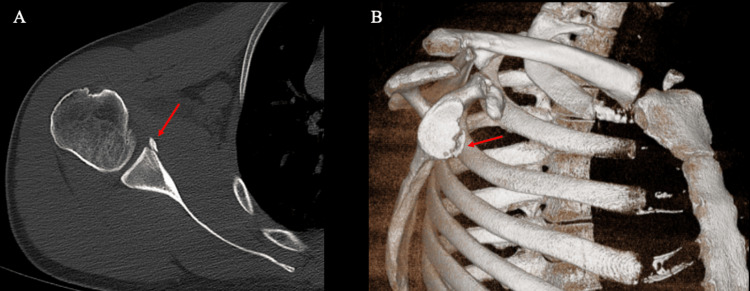
CT in the axial plane of the shoulder (Case 1) (A) glenoid fracture and bone loss, and (B) a 3D reconstruction indicating of the shoulder demonstrating anteroinferior glenoid fracture and bone loss. CT: computed tomography; 3D: three-dimensional

**Figure 2:**
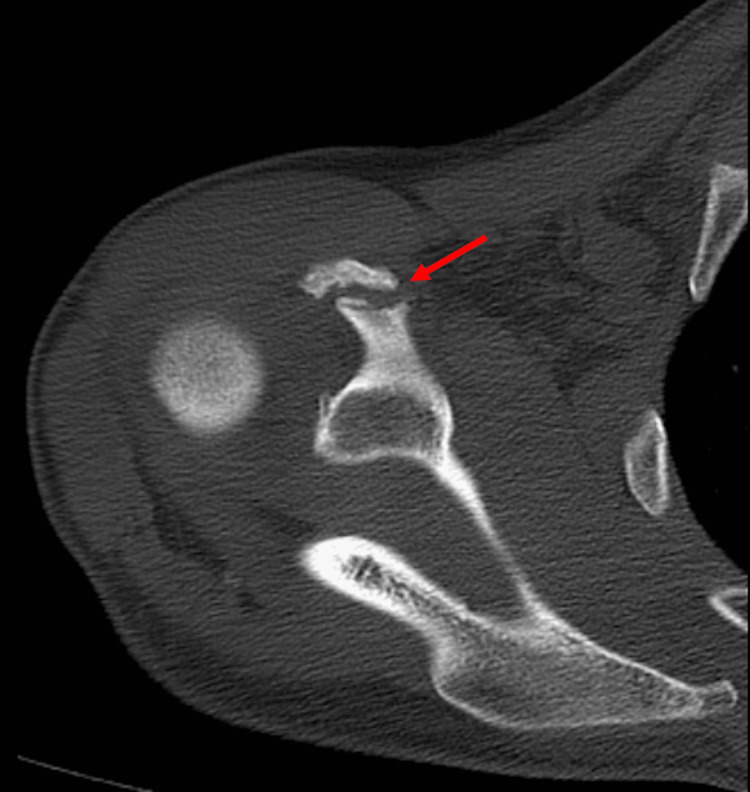
Axial CT scan of the right shoulder (Case 1) The red arrow shows coracoid non-union. CT: computed tomography

**Figure 3:**
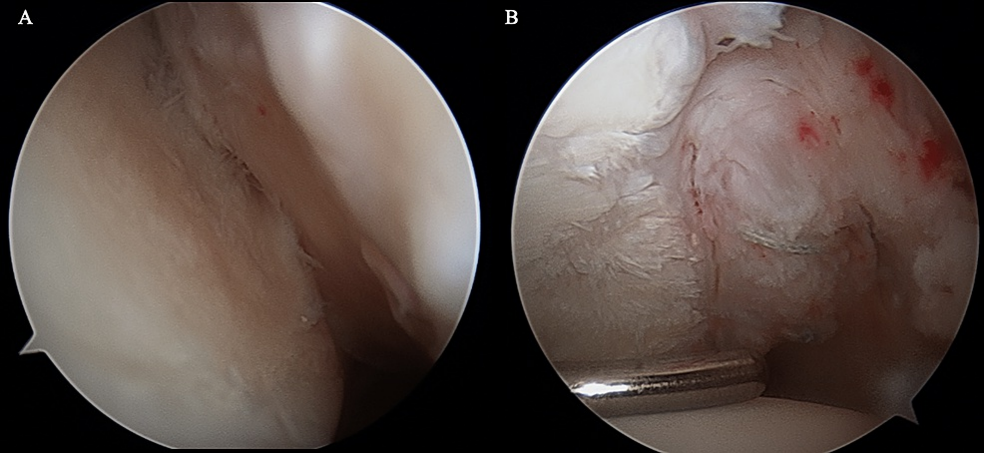
Arthroscopic view (Case 1) Engaging Hill Sachs lesion (A) and glenoid fragment (B).

A 30-year-old male presented with right shoulder pain after sustaining repeated shoulder dislocations from motocross accidents, despite previous arthroscopic Bankart repair. Imaging displayed a glenoid fracture with glenoid bone loss (Figure 1). A coracoid fracture was also noted near the tip of the coracoid, making him a poor candidate for a Latarjet (Figure 2). Treatment options were discussed, including glenoid augmentation. He elected to proceed with an arthroscopic remplissage and an open Bankart repair of both the fractured glenoid and the labrum, but without bony augmentation (Figure 3). His shoulder was stable at the end of the case and remained stable on exam throughout the rehabilitation process, which was uncomplicated. He returned to motocross despite understanding the risks of further injury.

Seven months after his second operation, he had another shoulder dislocation while racing motocross. He had several episodes of instability before presenting to the clinic. Physical examination was positive for apprehension and relocation testing. On MRI, the anterior glenoid bone loss and non-union of his coracoid remained present; at this point, the glenoid bone loss was about 1 cm and 25%-30% of the diameter of the articular surface. We discussed that further surgery was indicated and strongly recommend glenoid bone augmentation. It was discussed that his coracoid non-union would complicate a Latarjet procedure. We reviewed the risks and benefits of other grafts, including iliac crest autograft, distal clavicle autograft, distal tibial allograft, and scapular spine autograft. The patient chose surgical intervention using scapular spine autograft.

Under general anesthesia, the patient was placed into a beach chair position with the right upper extremity exposed. A deltopectoral incision was made over the anterior shoulder followed by dissection down to the clavipectoral fascia. The subscapularis tendon was visualized to perform a subscapularis split. The tendon was then bluntly dissected away from the capsule. With the underlying capsule exposed, a T-shaped incision was made, while leaving a small cuff of tissue attached to the humeral head to help facilitate repair during closure. Each limb of the capsule was tagged with sutures for later repair. The anterior glenoid neck and the glenoid surface were exposed and prepared as a flat bony bed. The defect was measured to be 1 cm in an anterior-to-posterior direction, which was consistent with the MRI interpretation.

**Figure 4:**
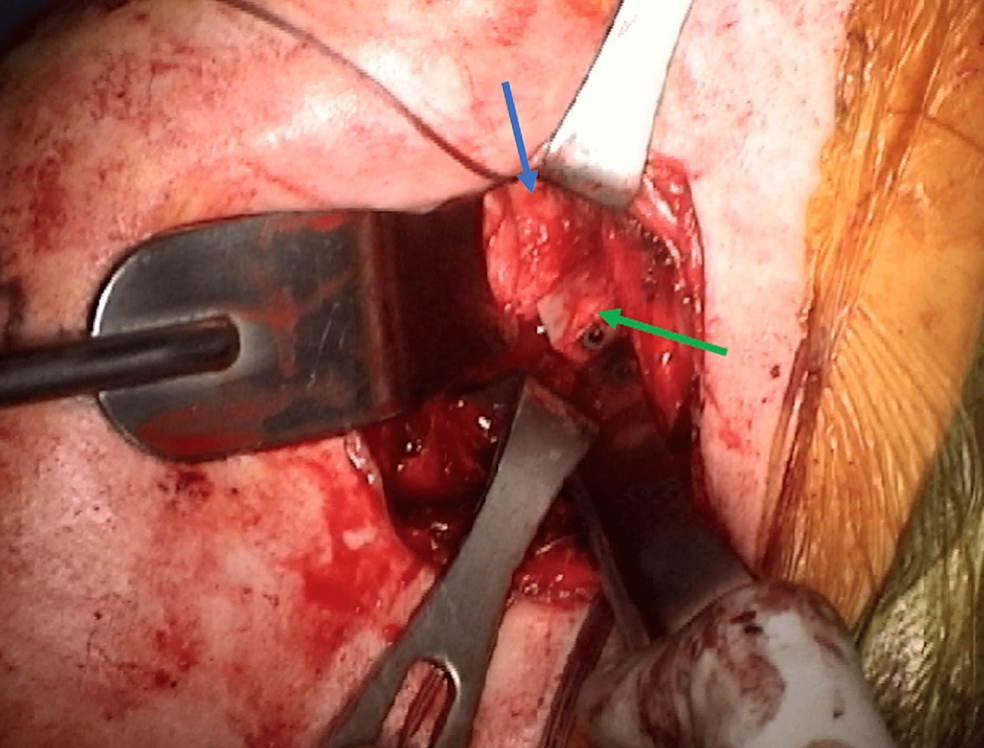
Scapular spine fixed in glenoid defect and flush with the glenoid surface in Case 1 The blue arrow indicates the glenoid, while the green arrow indicates the scapular spine fragment with one of two screws adjacent to the arrowhead.

To harvest the scapular spine graft, a horizontal incision was made over the spinous flare followed by sharp dissection down to the posterior bone of the spine. The fascia and periosteum were freed superiorly and inferiorly in full-thickness fashion to facilitate later repair. An oscillating saw was advanced vertically through the spine in two locations along the flare 2 cm apart to the base of the spine without penetrating the body of the scapula. The location of the flare was chosen based on maximizing scapular spine width while maintaining adequate distance from the spinoglenoid notch [4]. A curved osteotome was used to free the graft. The dimensions of the graft measured to be 1.5 cm in width, 2 cm in length, and 1 cm in depth. The graft was inspected and matched with the glenoid defect before being seated and drilled flush with the glenoid articular surface using the same technique as for a Latarjet procedure (Figure 4). The arm was taken through a range of motion, including 90 degrees of abduction with external rotation. There was no instability in any plane. The inferior and superior capsules were reefed, and the subscapularis split was closed. The shoulder was again evaluated with the arm in all provocative positions. The shoulder was stable with a functional range of motion on forward flexion, abduction, and external rotation, with only a 10-degree decrease in internal rotation.

**Figure 5:**
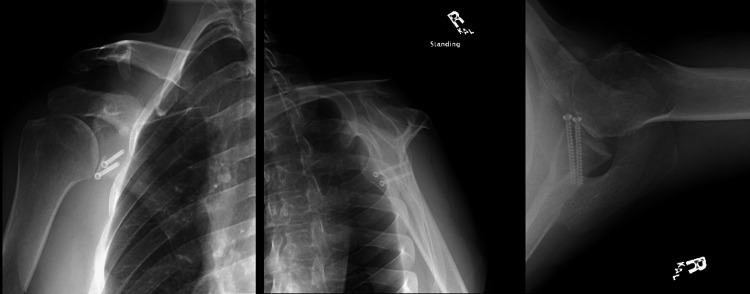
Radiographs of the right shoulder at three-month follow-up from case 1

Postoperatively, the upper extremity was placed into a sling for six weeks. His therapy followed our Latarjet protocol. At two weeks, he felt no shoulder instability while doing pendulum exercises and noted improving pain. At six weeks, he was progressing well with physical therapy and began working on active range of motion. Radiographs showed the hardware was in the appropriate position. At three months, his function continued to improve; he had no instability, but he reported intermittent catching, aching, and soreness within his shoulder with no overt pain. Radiographs again demonstrated that the hardware was in an appropriate position (Figure 5). At six months, the catching and soreness had improved. His range of motion had continued to progress, but he still experienced limitations relative to his left shoulder in internal and external rotations when his shoulder was abducted to 90 degrees; 10 degrees less in external rotation and 20 degrees less in internal rotation. He completed an upper extremity functional test with physical therapy and was discharged to a home exercise program. He was allowed a gradual return to unrestricted activity. We had a conversation about motocross and he planned to return, despite understanding the risks.

At 32 months, he reports that he competed in two full motocross seasons and is doing everything he’d like to do without functional limitations.

Case 2

**Figure 6:**
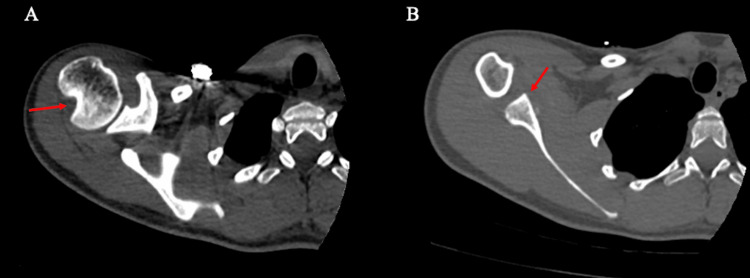
Axial CT scan (case 2) (A) large Hill-Sachs lesion and (B) glenoid bone loss CT: computed tomography

A 17-year-old male presented with right shoulder instability after requiring a reduction from a seizure episode. He had been diagnosed as being on the autism spectrum and having mild cerebral palsy. He was started on an anticonvulsant for his seizures and had no seizure activity for five months. It was unclear how much instability he was having during this time. The parents were worried about his shoulder being at risk due to his frequent episodes of flapping in addition to the seizure activity. Additionally, his mother noted that he had become less active with the right upper extremity since experiencing instability. A CT scan revealed a large Hill Sachs lesion and glenoid bone loss (Figure 6). Multiple options for glenoid augmentation were discussed, including open Latarjet, scapular spine autograft, and osteochondral allograft transplantation. Since it was not yet clear if his seizure activity was under control, we discussed issues for each graft choice if he did have further seizure activity. To preserve a Latarjet as an option once more time had passed to allow certainty about control of his seizure activity, we made the choice to use a scapular spine autograft. In addition to the bony procedures, arthroscopic remplissage was discussed.

**Figure 7:**
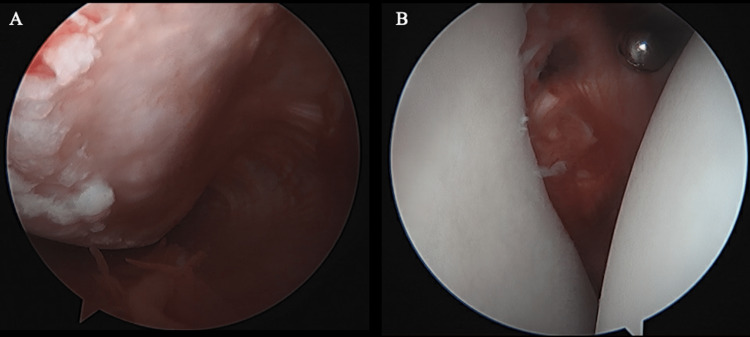
Arthroscopic view in Case 2 (A) Hill Sachs lesion and humeral head chondral damage, and (B) glenoid bone loss

The patient was positioned the same as in Case 1 above. Diagnostic arthroscopy demonstrated a large Hill Sachs lesion with osteochondral damage (Figure 7). There was an absent anteroinferior labrum on the glenoid surface and glenoid bone loss of 7 mm in an anterior-to-posterior direction. Arthroscopic remplissage was performed. Open glenoid augmentation with scapular spine autograft was then performed using the same technique as described in Case 1. The dimensions of the graft measured to be 1 cm in width, 2 cm in length, and 1 cm in depth. On post-operative exam, the shoulder was stable in all provocative positions. The range of motion was functional, but there was a 15-degree decrease in internal rotation, similar to Case 1.

**Figure 8:**
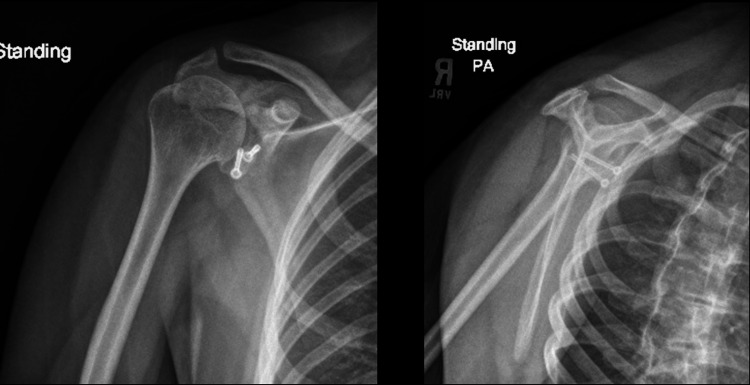
Radiographs of the right shoulder at five-week follow-up from Case 2

Postoperatively, the upper extremity was placed into a sling for six weeks. He was placed into our Latarjet therapy protocol. At two weeks, he felt no shoulder instability and passive exercises progressed. At five weeks, he was progressing well with physical therapy, specifically his range of motion. Radiographs showed the hardware was in the appropriate position (Figure 8). At five months, his function continued to improve, with no apprehension on examination. With the arm in 90 degrees abduction, external rotation was 15 degrees less than the other side with no other differences in the range of motion. At 20 months, his family reports that he is doing everything he’d like to do with no functional limitations and no further episodes of shoulder instability; his seizure activity has been well controlled.

## Discussion

Proper management of recurrent anterior shoulder instability for patients with significant osseous defects can be complex with several graft options available that each has inherent positives and negatives relative to one another. Patients who experience shoulder instability are often treated by arthroscopic Bankart repair, a generally effective procedure [7]. Assessment of the glenoid is important in determining the appropriate surgical treatment, as complex anterior shoulder instability with significant bone loss can cause high failure rates of arthroscopic Bankart repair [2,8]. In the presence of glenoid bone loss requiring intervention, the Latarjet technique is commonly performed [9]. Due to relatively high complication and reoperation rates with this technique, other options are continuing to be explored [5,10].

Alternative bone grafts presented in the literature for glenoid bone loss include coracoid, iliac crest, and distal tibia grafts [8,11-13]. More recently, the scapular spine has been studied as an alternative glenoid bone graft option [4-6]. With dimensions similar to the coracoid and iliac crest, the scapular spine has been reported to be a structurally suitable candidate for a glenoid bone graft [4]. One potential advantage of the scapular spine is the anatomic accessibility of the scapular spine making it possible to harvest the graft in the same surgical field as the rest of the procedure. Another potential advantage is the minimal donor site morbidity as the musculature around the harvest site remains overall intact and functional. While graft resorption is a possibility with any graft, the fact that the scapular spine is autologous may help limit this as well as other complications.

Both patients presented in this case series regained full function of their shoulder without any major complications to date, suggesting that glenoid augmentation with scapular spine autograft is a viable option for patients suffering from anterior shoulder instability due to glenoid bone loss. This procedure is an especially attractive option if there are contraindications to performing the Latarjet procedure or if there are circumstances when other options may be less appealing. There are limitations to the thickness of the graft that can be acquired given the depth of the scapular spine, therefore the amount of glenoid bone loss should be considered. With that said, studies have suggested that up to an average of 26% of glenoid bone loss can be adequately treated with scapular spine autograft [3]. Scapular spine autograft for glenoid bone loss should be considered as an option alongside other treatments for glenoid bone loss after adequate assessment of percent bone loss.

## Conclusions

Anterior shoulder dislocation injuries can progress to recurrent shoulder instability with osseous defects of the glenohumeral joint. There are various autograft and allograft options for glenoid augmentation to treat patients with glenoid defects. Open glenoid augmentation with scapular spine autograft has been demonstrated to provide adequate bone quality and biomechanics for smaller glenoid defects that require augmentation. The two cases presented here demonstrate that good clinical outcomes can also be achieved by utilizing this technique. Scapular spine autograft for glenoid augmentation should be considered as an option, along with other glenoid augmentation graft options.
